# Crystal structure of 2-phenyl-2λ^4^,3-ditellura­tetra­cyclo­[5.5.2.0^4,13^.0^10,14^]tetra­deca-1(12),4,6,10,13-pentaen-2-ylium tri­fluoro­methane­sulfonate

**DOI:** 10.1107/S1600536814018170

**Published:** 2014-08-13

**Authors:** Louise M. Diamond, Alexandra M. Z. Slawin, J. Derek Woollins

**Affiliations:** a EaStCHEM School of Chemistry, University of St Andrews, St Andrews, Fife KY16 9ST, Scotland

**Keywords:** crystal structure, acenaphthene, triflate, tellurium

## Abstract

In the title compound, C_18_H_13_Te_2_^+^·CF_3_O_3_S^−^, the Te^II^ atom of the cation and one O atom of the tri­fluoro­methane­sulfonate counter-ion form a close-to-linear Te—Te—O system, with a Te—Te—O angle of 172.3 (1)° and a Te—O distance of 2.816 (5) Å, which may suggest the presence of a three-centre–four-electron (3c–4e) bond. Secondary Te⋯O inter­actions [3.003 (4) and 3.016 (4) Å], involving the second Te^II^ atom of the binuclear mol­ecule, are also noted, resulting in a supra­molecular layer in the *bc* plane.

## Related literature   

For studies on related inter­actions with halogen counter-ions, see: Knight *et al.* (2010 ▶, 2012 ▶). For discussions of 3c–4e bonding in this type of system, see: Aschenbach *et al.* (2012 ▶). For a general review of *peri*-substituted naphthalenes and acenaphthenes, see: Kilian *et al.* (2011 ▶).
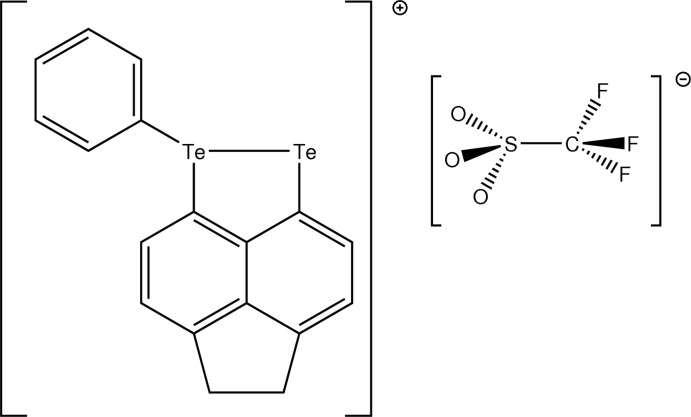


## Experimental   

### Crystal data  

C_18_H_13_Te_2_^+^·CF_3_O_3_S^−^
*M* *_r_* = 633.57
Monoclinic, 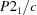

*a* = 10.687 (2) Å
*b* = 15.264 (3) Å
*c* = 12.242 (3) Å
β = 102.808 (6)°
*V* = 1947.4 (7) Å^3^
*Z* = 4
Mo *K*α radiation
μ = 3.15 mm^−1^
*T* = 93 K
0.06 × 0.03 × 0.03 mm

### Data collection  

Rigaku Mercury70 diffractometer
Absorption correction: multi-scan (*REQAB*; Rigaku, 1998 ▶) *T*_min_ = 0.619, *T*_max_ = 0.910
11783 measured reflections
3407 independent reflections
2926 reflections with *F* ^2^ > 2σ(*F* ^2^)
*R*_int_ = 0.053

### Refinement  

*R*[*F* ^2^ > 2σ(*F* ^2^)] = 0.041
*wR*(*F* ^2^) = 0.100
*S* = 1.07
3407 reflections
253 parameters
H-atom parameters constrained
Δρ_max_ = 1.19 e Å^−3^
Δρ_min_ = −1.03 e Å^−3^

### 

Data collection: *CrystalClear-SM Expert* (Rigaku, 2009 ▶); cell refinement: *CrystalClear-SM Expert*; data reduction: *CrystalClear-SM Expert*; program(s) used to solve structure: *SHELXS97* (Sheldrick, 2008 ▶); program(s) used to refine structure: *SHELXL2013* (Sheldrick, 2008 ▶); molecular graphics: *CrystalStructure* (Rigaku, 2014 ▶); software used to prepare material for publication: *CrystalStructure*.

## Supplementary Material

Crystal structure: contains datablock(s) global, I. DOI: 10.1107/S1600536814018170/tk5334sup1.cif

Structure factors: contains datablock(s) I. DOI: 10.1107/S1600536814018170/tk5334Isup2.hkl

Supporting information file. DOI: 10.1107/S1600536814018170/tk5334Isup3.cdx

Supporting information file. DOI: 10.1107/S1600536814018170/tk5334Isup4.cml

**Fig. 1.**
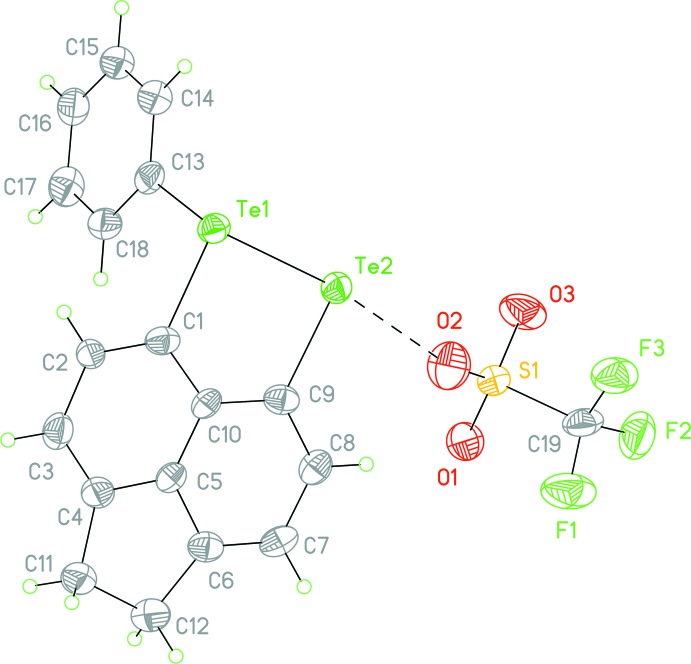
The molecular structure of I with displacement ellipsoids drawn at the 50% probability level, hydrogen atoms omitted for clarity.

. DOI: 10.1107/S1600536814018170/tk5334fig1.tif
The mol­ecular structure of I with displacement ellipsoids drawn at the 50% probability level, hydrogen atoms omitted for clarity.

CCDC reference: 1018417

Additional supporting information:  crystallographic information; 3D view; checkCIF report

## Figures and Tables

**Table 1:** Selected bond lengths (Å)

Te1—Te2	2.7297 (6)
Te1—C1	2.104 (5)
Te1—C13	2.130 (6)
Te2—C9	2.131 (5)

## supplementary crystallographic information

### S1.1. Synthesis and crystallization

5,6-Bis(phenyl­telluro)acenaphthene (0.22 g, 0.39 mmol) was added to a solution of copper triflate (0.15 g, 0.40 mmol) in di­chloro­methane (20 ml) at 263 K. The resulting dark-purple solution was left to stir at this temperature for 3 h, then at room temperature for a further 12 h. The solution was filtered to give a grey solid and a dark-orange filtrate. The filtrate was evaporated under reduced pressure to yield an orange oil. The oil was redissolved in the minimum amount of di­chloro­methane, hexane was layered on top and the solution was left at 243 K. After 24 h the solution had yielded red needle-like crystals (0.03 g, 9%).

### S1.2. Refinement

Carbon-bound H-atoms were placed in calculated positions (C—H = 0.95–0.99 Å) and were included in the refinement in the riding model approximation, with *U*_iso_(H) set to 1.2*U*_equiv_(C). The highest peak in the difference map is 1.06 Å from atom H7.

### S2. Results and discussion

ENTER TEXT

### Crystal data

**Table:**

C_18_H_13_Te_2_^+^·CF_3_O_3_S^−^	*F*(000) = 1192.00
*M**_r_* = 633.57	*D*_x_ = 2.161 Mg m^−^^3^
Monoclinic, *P*2_1_/*c*	Mo *K*α radiation, λ = 0.71075 Å
Hall symbol: -P 2ybc	Cell parameters from 6711 reflections
*a* = 10.687 (2) Å	θ = 2.2–25.4°
*b* = 15.264 (3) Å	µ = 3.15 mm^−^^1^
*c* = 12.242 (3) Å	*T* = 93 K
β = 102.808 (6)°	Prism, red
*V* = 1947.4 (7) Å^3^	0.06 × 0.03 × 0.03 mm
*Z* = 4	

### Data collection

**Table:**

Rigaku Mercury70 diffractometer	2926 reflections with *F*^2^ > 2σ(*F*^2^)
Detector resolution: 14.629 pixels mm^-1^	*R*_int_ = 0.053
ω scans	θ_max_ = 25.0°, θ_min_ = 2.2°
Absorption correction: multi-scan (*REQAB*; Rigaku, 1998)	*h* = −12→9
*T*_min_ = 0.619, *T*_max_ = 0.910	*k* = −18→16
11783 measured reflections	*l* = −13→14
3407 independent reflections	

### Refinement

**Table:**

Refinement on *F*^2^	Secondary atom site location: difference Fourier map
*R*[*F*^2^ > 2σ(*F*^2^)] = 0.041	Hydrogen site location: inferred from neighbouring sites
*wR*(*F*^2^) = 0.100	H-atom parameters constrained
*S* = 1.07	*w* = 1/[σ^2^(*F*_o_^2^) + (0.0442*P*)^2^ + 0.2589*P*] where *P* = (*F*_o_^2^ + 2*F*_c_^2^)/3
3407 reflections	(Δ/σ)_max_ = 0.001
253 parameters	Δρ_max_ = 1.19 e Å^−^^3^
0 restraints	Δρ_min_ = −1.03 e Å^−^^3^
Primary atom site location: structure-invariant direct methods	

### Special details

**Table:**

Geometry. ENTER SPECIAL DETAILS OF THE MOLECULAR GEOMETRY
Refinement. Refinement was performed using all reflections. The weighted *R*-factor (*wR*) and goodness of fit (*S*) are based on *F*^2^. *R*-factor (gt) are based on *F*. The threshold expression of *F*^2^ > 2.0 σ(*F*^2^) is used only for calculating *R*-factor (gt).

### Fractional atomic coordinates and isotropic or equivalent isotropic displacement parameters (Å^2^)

**Table:**

	*x*	*y*	*z*	*U*_iso_*/*U*_eq_	
Te1	0.92263 (3)	0.03268 (2)	0.27873 (3)	0.03050 (15)	
Te2	1.06486 (3)	0.15215 (2)	0.19281 (3)	0.03058 (15)	
S1	1.22291 (14)	0.36194 (10)	0.05449 (12)	0.0343 (3)	
F1	1.3368 (4)	0.2922 (3)	−0.0924 (3)	0.0736 (12)	
F2	1.4264 (3)	0.4088 (3)	−0.0175 (3)	0.0663 (11)	
F3	1.4490 (3)	0.2900 (3)	0.0760 (3)	0.0580 (10)	
O1	1.1412 (4)	0.4067 (3)	−0.0381 (3)	0.0504 (11)	
O2	1.1831 (5)	0.2769 (3)	0.0794 (4)	0.0708 (15)	
O3	1.2729 (4)	0.4160 (3)	0.1503 (3)	0.0561 (12)	
C1	0.8098 (5)	0.0140 (3)	0.1162 (4)	0.0306 (12)	
C2	0.7081 (5)	−0.0459 (4)	0.0972 (4)	0.0336 (13)	
C3	0.6348 (5)	−0.0581 (4)	−0.0142 (5)	0.0369 (13)	
C4	0.6668 (5)	−0.0150 (3)	−0.1026 (5)	0.0339 (13)	
C5	0.7726 (5)	0.0419 (3)	−0.0806 (4)	0.0254 (12)	
C6	0.7958 (5)	0.0781 (3)	−0.1800 (4)	0.0323 (12)	
C7	0.8986 (6)	0.1343 (4)	−0.1715 (5)	0.0348 (13)	
C8	0.9751 (6)	0.1531 (3)	−0.0650 (5)	0.0323 (13)	
C9	0.9514 (5)	0.1190 (3)	0.0320 (4)	0.0311 (12)	
C10	0.8473 (5)	0.0597 (3)	0.0259 (4)	0.0272 (11)	
C11	0.6098 (6)	−0.0171 (4)	−0.2265 (5)	0.0393 (14)	
C12	0.6954 (6)	0.0459 (4)	−0.2789 (5)	0.0398 (14)	
C13	0.8004 (5)	0.1217 (3)	0.3394 (5)	0.0321 (12)	
C14	0.8276 (6)	0.1392 (4)	0.4527 (5)	0.0383 (14)	
C15	0.7547 (5)	0.1992 (4)	0.4949 (5)	0.0378 (13)	
C16	0.6548 (5)	0.2397 (4)	0.4255 (5)	0.0374 (13)	
C17	0.6254 (5)	0.2217 (4)	0.3126 (5)	0.0405 (14)	
C18	0.6985 (5)	0.1620 (3)	0.2671 (5)	0.0341 (13)	
C19	1.3656 (6)	0.3386 (4)	0.0029 (5)	0.0411 (15)	
H2	0.68829	−0.07808	0.15763	0.0403*	
H3	0.56297	−0.09649	−0.02735	0.0443*	
H7	0.91742	0.15997	−0.23669	0.0417*	
H8	1.04654	0.19114	−0.06005	0.0387*	
H11A	0.51973	0.00346	−0.24279	0.0472*	
H11B	0.61218	−0.07721	−0.25642	0.0472*	
H12A	0.73565	0.01444	−0.33292	0.0478*	
H12B	0.64423	0.09538	−0.31778	0.0478*	
H14	0.89646	0.10994	0.50143	0.0460*	
H15	0.77437	0.21228	0.57267	0.0453*	
H16	0.60456	0.28089	0.45527	0.0449*	
H17	0.55472	0.25015	0.26522	0.0486*	
H18	0.67911	0.14935	0.18917	0.0409*	

### Atomic displacement parameters (Å^2^)

**Table:**

	*U*^11^	*U*^22^	*U*^33^	*U*^12^	*U*^13^	*U*^23^
Te1	0.0320 (2)	0.0332 (3)	0.0270 (2)	0.00187 (14)	0.00796 (17)	0.00255 (15)
Te2	0.0295 (3)	0.0324 (3)	0.0307 (2)	−0.00155 (14)	0.00854 (18)	−0.00344 (15)
S1	0.0339 (8)	0.0419 (8)	0.0283 (8)	−0.0023 (6)	0.0093 (6)	−0.0031 (6)
F1	0.073 (3)	0.106 (3)	0.040 (2)	0.035 (3)	0.009 (2)	−0.018 (2)
F2	0.043 (2)	0.092 (3)	0.066 (3)	−0.004 (2)	0.0170 (19)	0.030 (2)
F3	0.055 (2)	0.080 (3)	0.038 (2)	0.022 (2)	0.0064 (17)	0.0081 (19)
O1	0.033 (2)	0.075 (3)	0.043 (2)	0.015 (2)	0.0056 (19)	0.005 (2)
O2	0.075 (3)	0.069 (3)	0.069 (3)	−0.039 (3)	0.019 (3)	0.007 (3)
O3	0.056 (3)	0.068 (3)	0.041 (2)	0.011 (2)	0.004 (2)	−0.026 (2)
C1	0.035 (3)	0.033 (3)	0.024 (3)	0.006 (2)	0.007 (2)	−0.001 (2)
C2	0.025 (3)	0.049 (3)	0.028 (3)	−0.003 (2)	0.008 (2)	−0.015 (3)
C3	0.034 (3)	0.038 (3)	0.043 (3)	−0.009 (2)	0.015 (3)	−0.008 (3)
C4	0.032 (3)	0.040 (3)	0.030 (3)	0.001 (2)	0.008 (3)	−0.008 (3)
C5	0.029 (3)	0.022 (3)	0.029 (3)	0.004 (2)	0.012 (2)	−0.002 (2)
C6	0.038 (3)	0.031 (3)	0.028 (3)	0.003 (2)	0.007 (2)	−0.003 (2)
C7	0.047 (4)	0.032 (3)	0.029 (3)	0.005 (3)	0.016 (3)	0.006 (2)
C8	0.038 (3)	0.025 (3)	0.037 (3)	−0.002 (2)	0.015 (3)	0.002 (2)
C9	0.036 (3)	0.029 (3)	0.027 (3)	0.005 (2)	0.005 (2)	−0.002 (2)
C10	0.029 (3)	0.028 (3)	0.028 (3)	0.003 (2)	0.014 (2)	−0.003 (2)
C11	0.041 (3)	0.044 (3)	0.032 (3)	−0.008 (3)	0.007 (3)	−0.006 (3)
C12	0.048 (4)	0.039 (3)	0.033 (3)	−0.001 (3)	0.010 (3)	−0.001 (3)
C13	0.030 (3)	0.035 (3)	0.032 (3)	−0.004 (2)	0.011 (2)	0.006 (3)
C14	0.037 (3)	0.043 (3)	0.035 (3)	−0.003 (3)	0.008 (3)	−0.002 (3)
C15	0.038 (3)	0.043 (3)	0.033 (3)	−0.006 (3)	0.009 (3)	−0.003 (3)
C16	0.033 (3)	0.038 (3)	0.043 (3)	−0.003 (3)	0.012 (3)	−0.004 (3)
C17	0.035 (3)	0.039 (3)	0.048 (4)	0.008 (3)	0.009 (3)	0.011 (3)
C18	0.035 (3)	0.038 (3)	0.029 (3)	0.001 (2)	0.006 (3)	0.004 (2)
C19	0.047 (4)	0.053 (4)	0.023 (3)	0.010 (3)	0.008 (3)	0.008 (3)

### Geometric parameters (Å, º)

**Table:**

Te1—Te2	2.7297 (6)	C8—C9	1.370 (8)
Te1—C1	2.104 (5)	C9—C10	1.424 (7)
Te1—C13	2.130 (6)	C11—C12	1.560 (9)
Te2—C9	2.131 (5)	C13—C14	1.379 (8)
S1—O1	1.441 (4)	C13—C18	1.385 (7)
S1—O2	1.420 (5)	C14—C15	1.374 (8)
S1—O3	1.437 (4)	C15—C16	1.357 (7)
S1—C19	1.810 (7)	C16—C17	1.376 (8)
F1—C19	1.341 (7)	C17—C18	1.394 (8)
F2—C19	1.307 (7)	C2—H2	0.950
F3—C19	1.339 (6)	C3—H3	0.950
C1—C2	1.401 (7)	C7—H7	0.950
C1—C10	1.437 (8)	C8—H8	0.950
C2—C3	1.426 (7)	C11—H11A	0.990
C3—C4	1.373 (8)	C11—H11B	0.990
C4—C5	1.403 (7)	C12—H12A	0.990
C4—C11	1.505 (7)	C12—H12B	0.990
C5—C6	1.408 (8)	C14—H14	0.950
C5—C10	1.396 (7)	C15—H15	0.950
C6—C7	1.379 (8)	C16—H16	0.950
C6—C12	1.510 (7)	C17—H17	0.950
C7—C8	1.407 (7)	C18—H18	0.950
			
F1···O1	2.913 (6)	F2···H11A^xiv^	3.5516
F1···O2	2.951 (7)	F2···H11B^vi^	2.7142
F2···O1	3.003 (5)	F2···H12A^vi^	2.7467
F2···O3	2.902 (6)	F2···H12B^vi^	3.4740
F3···O2	2.859 (6)	F2···H12B^xiv^	2.9799
F3···O3	2.972 (6)	F2···H16^iv^	3.5216
C1···C4	2.810 (7)	F3···H3^ix^	3.0105
C1···C18	3.302 (8)	F3···H12B^xiv^	2.8107
C2···C5	2.770 (8)	F3···H15^iv^	3.4861
C3···C6	3.600 (8)	F3···H16^iv^	2.6844
C3···C10	2.853 (7)	F3···H17^vii^	2.4194
C5···C8	2.724 (7)	F3···H18^vii^	3.3265
C6···C9	2.825 (7)	O1···H8	3.4347
C7···C10	2.832 (8)	O1···H12A^vi^	2.8026
C13···C16	2.739 (8)	O1···H14^viii^	3.1733
C14···C17	2.748 (7)	O1···H14^iii^	2.7746
C15···C18	2.778 (8)	O2···H8	2.3778
Te1···O1^i^	3.003 (4)	O2···H14^iii^	3.4610
Te1···O3^ii^	3.016 (5)	O3···H2^viii^	2.2975
Te2···F1^i^	3.563 (4)	O3···H11A^xiv^	2.9415
Te2···O1^i^	3.336 (4)	C2···H3^x^	3.5714
Te2···O2	2.816 (5)	C2···H8^ix^	3.5384
F1···Te2^iii^	3.563 (4)	C2···H11A^x^	3.3843
F1···C16^iv^	3.391 (7)	C3···H3^x^	3.2820
F1···C17^iv^	3.536 (8)	C5···H16^iii^	3.3296
F2···F2^v^	3.179 (6)	C7···H15^xv^	3.3385
F2···C11^vi^	3.272 (7)	C9···H15^iii^	3.2977
F2···C12^vi^	3.294 (7)	C10···H16^iii^	3.5217
F2···C16^iv^	3.512 (7)	C12···H15^xv^	3.3381
F3···C16^iv^	3.198 (7)	C12···H17^iii^	3.5496
F3···C17^vii^	3.258 (6)	C14···H8^i^	3.5166
O1···Te1^iii^	3.003 (4)	C14···H12A^xvi^	3.5530
O1···Te2^iii^	3.336 (4)	C15···H7^xvi^	3.4127
O1···C14^iii^	3.401 (7)	C15···H12A^xvi^	3.5535
O2···Te2	2.816 (5)	C15···H12B^xvi^	3.2196
O2···C8	3.145 (7)	C15···H18^i^	3.5368
O2···C9	3.411 (7)	C17···H11B^x^	3.3179
O3···Te1^viii^	3.016 (5)	C17···H12B^i^	3.2449
O3···C1^viii^	3.509 (7)	C18···H7^i^	3.5931
O3···C2^viii^	3.108 (7)	C18···H11A^x^	3.4055
O3···C13^viii^	3.245 (7)	C18···H11B^x^	3.5389
C1···O3^ii^	3.509 (7)	C18···H15^iii^	3.2957
C1···C8^ix^	3.581 (8)	C19···H11B^vi^	3.3364
C2···O3^ii^	3.108 (7)	C19···H12A^vi^	3.4216
C3···C3^x^	3.466 (8)	C19···H12B^xiv^	3.4361
C5···C16^iii^	3.571 (7)	C19···H16^iv^	3.2939
C6···C16^iii^	3.542 (8)	H2···S1^ii^	3.5604
C6···C17^iii^	3.549 (8)	H2···F1^ix^	3.3609
C8···O2	3.145 (7)	H2···O3^ii^	2.2975
C8···C1^ix^	3.581 (8)	H2···H11A^x^	2.8904
C8···C14^iii^	3.568 (8)	H3···F1^ix^	3.3946
C8···C15^iii^	3.455 (8)	H3···F3^ix^	3.0105
C9···O2	3.411 (7)	H3···C2^x^	3.5714
C9···C15^iii^	3.450 (7)	H3···C3^x^	3.2820
C11···F2^xi^	3.272 (7)	H3···H3^x^	3.3670
C12···F2^xi^	3.294 (7)	H3···H16^xvii^	2.8615
C13···O3^ii^	3.245 (7)	H3···H18^x^	2.9994
C14···O1^i^	3.401 (7)	H7···Te1^ix^	3.4963
C14···C8^i^	3.568 (8)	H7···Te2^iii^	3.4720
C15···C8^i^	3.455 (8)	H7···C15^xv^	3.4127
C15···C9^i^	3.450 (7)	H7···C18^iii^	3.5931
C16···F1^xii^	3.391 (7)	H7···H14^xv^	3.2547
C16···F2^xii^	3.512 (7)	H7···H15^xv^	2.6153
C16···F3^xii^	3.198 (7)	H8···S1	3.3383
C16···C5^i^	3.571 (7)	H8···F1	3.5644
C16···C6^i^	3.542 (8)	H8···O1	3.4347
C17···F1^xii^	3.536 (8)	H8···O2	2.3778
C17···F3^xiii^	3.258 (6)	H8···C2^ix^	3.5384
C17···C6^i^	3.549 (8)	H8···C14^iii^	3.5166
Te1···H2	3.1116	H8···H14^iii^	3.5907
Te1···H14	3.0397	H11A···F2^xi^	3.4316
Te1···H18	3.1435	H11A···F2^xviii^	3.5516
Te2···H8	3.1155	H11A···O3^xviii^	2.9415
C1···H3	3.2925	H11A···C2^x^	3.3843
C1···H18	2.7511	H11A···C18^x^	3.4055
C2···H18	3.2258	H11A···H2^x^	2.8904
C3···H11A	2.9500	H11A···H18^x^	3.3175
C3···H11B	2.9360	H11B···Te2^ix^	3.5548
C4···H2	3.2867	H11B···F1^xi^	2.8537
C4···H12A	3.1007	H11B···F2^xi^	2.7142
C4···H12B	3.0915	H11B···C17^x^	3.3179
C5···H3	3.2482	H11B···C18^x^	3.5389
C5···H7	3.2570	H11B···C19^xi^	3.3364
C5···H11A	3.0345	H11B···H17^x^	3.1743
C5···H11B	3.0394	H11B···H18^x^	3.5662
C5···H12A	3.0546	H12A···Te2^ix^	3.5117
C5···H12B	3.0352	H12A···F1^xi^	3.5539
C6···H8	3.2511	H12A···F2^xi^	2.7467
C6···H11A	3.0967	H12A···O1^xi^	2.8026
C6···H11B	3.0874	H12A···C14^xv^	3.5530
C7···H12A	2.9595	H12A···C15^xv^	3.5535
C7···H12B	2.9649	H12A···C19^xi^	3.4216
C9···H7	3.2884	H12A···H14^xv^	3.2766
C10···H2	3.3355	H12A···H15^xv^	3.2922
C10···H8	3.2652	H12B···F2^xi^	3.4740
C10···H18	3.2698	H12B···F2^xviii^	2.9799
C11···H3	2.8646	H12B···F3^xviii^	2.8107
C12···H7	2.8954	H12B···C15^xv^	3.2196
C13···H15	3.2397	H12B···C17^iii^	3.2449
C13···H17	3.2406	H12B···C19^xviii^	3.4361
C14···H16	3.2237	H12B···H15^xv^	2.7828
C14···H18	3.2682	H12B···H17^iii^	2.8174
C15···H17	3.2273	H14···S1^i^	3.4313
C16···H14	3.2238	H14···O1^ii^	3.1733
C16···H18	3.2674	H14···O1^i^	2.7746
C17···H15	3.2316	H14···O2^i^	3.4610
C18···H14	3.2628	H14···H7^xvi^	3.2547
C18···H16	3.2611	H14···H8^i^	3.5907
H2···H3	2.3783	H14···H12A^xvi^	3.2766
H2···H18	3.4967	H15···F3^xii^	3.4861
H3···H11A	2.9928	H15···C7^xvi^	3.3385
H3···H11B	2.9802	H15···C9^i^	3.2977
H7···H8	2.3418	H15···C12^xvi^	3.3381
H7···H12A	3.0135	H15···C18^i^	3.2957
H7···H12B	3.0326	H15···H7^xvi^	2.6153
H11A···H12A	2.7748	H15···H12A^xvi^	3.2922
H11A···H12B	2.2635	H15···H12B^xvi^	2.7828
H11B···H12A	2.2635	H15···H18^i^	2.8592
H11B···H12B	2.7813	H16···F1^xii^	3.0054
H14···H15	2.3255	H16···F2^xii^	3.5216
H15···H16	2.3021	H16···F3^xii^	2.6844
H16···H17	2.3168	H16···C5^i^	3.3296
H17···H18	2.3559	H16···C10^i^	3.5217
Te1···H7^ix^	3.4963	H16···C19^xii^	3.2939
Te2···H7^i^	3.4720	H16···H3^xix^	2.8615
Te2···H11B^ix^	3.5548	H16···H18^i^	2.9927
Te2···H12A^ix^	3.5117	H17···F1^xii^	3.2678
S1···H2^viii^	3.5604	H17···F3^xiii^	2.4194
S1···H8	3.3383	H17···C12^i^	3.5496
S1···H14^iii^	3.4313	H17···H11B^x^	3.1743
F1···H2^ix^	3.3609	H17···H12B^i^	2.8174
F1···H3^ix^	3.3946	H18···F3^xiii^	3.3265
F1···H8	3.5644	H18···C15^iii^	3.5368
F1···H11B^vi^	2.8537	H18···H3^x^	2.9994
F1···H12A^vi^	3.5539	H18···H11A^x^	3.3175
F1···H16^iv^	3.0054	H18···H11B^x^	3.5662
F1···H17^iv^	3.2678	H18···H15^iii^	2.8592
F2···H11A^vi^	3.4316	H18···H16^iii^	2.9927
			
Te2—Te1—C1	88.70 (15)	C14—C15—C16	120.1 (5)
Te2—Te1—C13	98.34 (15)	C15—C16—C17	120.7 (5)
C1—Te1—C13	98.6 (2)	C16—C17—C18	120.6 (5)
Te1—Te2—C9	86.91 (15)	C13—C18—C17	117.7 (5)
O1—S1—O2	116.3 (3)	S1—C19—F1	111.1 (4)
O1—S1—O3	114.9 (3)	S1—C19—F2	113.5 (4)
O1—S1—C19	103.3 (3)	S1—C19—F3	111.3 (4)
O2—S1—O3	115.1 (3)	F1—C19—F2	107.1 (5)
O2—S1—C19	102.2 (3)	F1—C19—F3	106.2 (5)
O3—S1—C19	102.0 (3)	F2—C19—F3	107.2 (5)
Te1—C1—C2	120.4 (4)	C1—C2—H2	120.450
Te1—C1—C10	117.6 (3)	C3—C2—H2	120.449
C2—C1—C10	121.8 (4)	C2—C3—H3	119.656
C1—C2—C3	119.1 (5)	C4—C3—H3	119.651
C2—C3—C4	120.7 (5)	C6—C7—H7	120.424
C3—C4—C5	118.5 (5)	C8—C7—H7	120.429
C3—C4—C11	131.9 (5)	C7—C8—H8	118.517
C5—C4—C11	109.6 (5)	C9—C8—H8	118.501
C4—C5—C6	111.5 (4)	C4—C11—H11A	110.757
C4—C5—C10	124.7 (5)	C4—C11—H11B	110.756
C6—C5—C10	123.8 (5)	C12—C11—H11A	110.759
C5—C6—C7	118.0 (4)	C12—C11—H11B	110.756
C5—C6—C12	109.5 (5)	H11A—C11—H11B	108.843
C7—C6—C12	132.5 (5)	C6—C12—H12A	110.873
C6—C7—C8	119.1 (5)	C6—C12—H12B	110.872
C7—C8—C9	123.0 (5)	C11—C12—H12A	110.874
Te2—C9—C8	122.3 (4)	C11—C12—H12B	110.871
Te2—C9—C10	118.5 (4)	H12A—C12—H12B	108.910
C8—C9—C10	119.2 (4)	C13—C14—H14	120.132
C1—C10—C5	115.2 (4)	C15—C14—H14	120.134
C1—C10—C9	128.0 (4)	C14—C15—H15	119.953
C5—C10—C9	116.9 (5)	C16—C15—H15	119.953
C4—C11—C12	104.9 (4)	C15—C16—H16	119.680
C6—C12—C11	104.4 (4)	C17—C16—H16	119.670
Te1—C13—C14	117.7 (4)	C16—C17—H17	119.695
Te1—C13—C18	121.1 (4)	C18—C17—H17	119.691
C14—C13—C18	121.2 (5)	C13—C18—H18	121.144
C13—C14—C15	119.7 (5)	C17—C18—H18	121.146
			
Te2—Te1—C1—C2	179.4 (3)	C3—C4—C11—C12	−178.6 (5)
Te2—Te1—C1—C10	4.1 (3)	C5—C4—C11—C12	0.4 (6)
C1—Te1—Te2—C9	−4.04 (15)	C11—C4—C5—C6	−1.5 (6)
Te2—Te1—C13—C14	−101.8 (3)	C11—C4—C5—C10	179.5 (4)
Te2—Te1—C13—C18	76.9 (4)	C4—C5—C6—C7	−178.9 (4)
C13—Te1—Te2—C9	−102.50 (13)	C4—C5—C6—C12	2.0 (6)
C1—Te1—C13—C14	168.2 (3)	C4—C5—C10—C1	0.7 (7)
C1—Te1—C13—C18	−13.0 (4)	C4—C5—C10—C9	179.7 (4)
C13—Te1—C1—C2	−82.3 (4)	C6—C5—C10—C1	−178.2 (4)
C13—Te1—C1—C10	102.3 (3)	C6—C5—C10—C9	0.8 (7)
Te1—Te2—C9—C8	−175.5 (4)	C10—C5—C6—C7	0.1 (7)
Te1—Te2—C9—C10	4.7 (3)	C10—C5—C6—C12	−179.0 (4)
O1—S1—C19—F1	−56.7 (4)	C5—C6—C7—C8	−0.1 (8)
O1—S1—C19—F2	64.1 (4)	C5—C6—C12—C11	−1.6 (5)
O1—S1—C19—F3	−174.8 (3)	C7—C6—C12—C11	179.4 (5)
O2—S1—C19—F1	64.5 (4)	C12—C6—C7—C8	178.8 (5)
O2—S1—C19—F2	−174.8 (3)	C6—C7—C8—C9	−1.0 (8)
O2—S1—C19—F3	−53.7 (4)	C7—C8—C9—Te2	−177.8 (4)
O3—S1—C19—F1	−176.2 (3)	C7—C8—C9—C10	2.0 (8)
O3—S1—C19—F2	−55.4 (4)	Te2—C9—C10—C1	−3.2 (7)
O3—S1—C19—F3	65.7 (4)	Te2—C9—C10—C5	177.9 (3)
Te1—C1—C2—C3	−179.0 (3)	C8—C9—C10—C1	177.0 (4)
Te1—C1—C10—C5	177.2 (3)	C8—C9—C10—C5	−1.8 (7)
Te1—C1—C10—C9	−1.7 (7)	C4—C11—C12—C6	0.7 (5)
C2—C1—C10—C5	2.0 (7)	Te1—C13—C14—C15	177.1 (3)
C2—C1—C10—C9	−176.9 (5)	Te1—C13—C18—C17	−177.9 (3)
C10—C1—C2—C3	−3.9 (8)	C14—C13—C18—C17	0.8 (8)
C1—C2—C3—C4	3.1 (8)	C18—C13—C14—C15	−1.6 (8)
C2—C3—C4—C5	−0.6 (8)	C13—C14—C15—C16	1.3 (8)
C2—C3—C4—C11	178.3 (5)	C14—C15—C16—C17	−0.3 (8)
C3—C4—C5—C6	177.6 (4)	C15—C16—C17—C18	−0.5 (8)
C3—C4—C5—C10	−1.4 (8)	C16—C17—C18—C13	0.3 (8)

Symmetry codes: (i) *x*, −*y*+1/2, *z*+1/2; (ii) −*x*+2, *y*−1/2, −*z*+1/2; (iii) *x*, −*y*+1/2, *z*−1/2; (iv) *x*+1, −*y*+1/2, *z*−1/2; (v) −*x*+3, −*y*+1, −*z*; (vi) −*x*+2, *y*+1/2, −*z*−1/2; (vii) *x*+1, *y*, *z*; (viii) −*x*+2, *y*+1/2, −*z*+1/2; (ix) −*x*+2, −*y*, −*z*; (x) −*x*+1, −*y*, −*z*; (xi) −*x*+2, *y*−1/2, −*z*−1/2; (xii) *x*−1, −*y*+1/2, *z*+1/2; (xiii) *x*−1, *y*, *z*; (xiv) *x*+1, −*y*+1/2, *z*+1/2; (xv) *x*, *y*, *z*−1; (xvi) *x*, *y*, *z*+1; (xvii) −*x*+1, *y*−1/2, −*z*+1/2; (xviii) *x*−1, −*y*+1/2, *z*−1/2; (xix) −*x*+1, *y*+1/2, −*z*+1/2.
