# Supplementary figures and images for: Crystal structure of 2-phenyl-2λ4,3-ditellura­tetra­cyclo­[5.5.2.04,13.010,14]tetra­deca-1(12),4,6,10,13-pentaen-2-ylium tri­fluoro­methane­sulfonate
Source: Acta Crystallogr Sect E Struct Rep Online. 2014 Aug 13;70(Pt 9):o1003–4. doi: 10.1107/S1600536814018170 (PMC4186063; doi:10.1107/S1600536814018170)

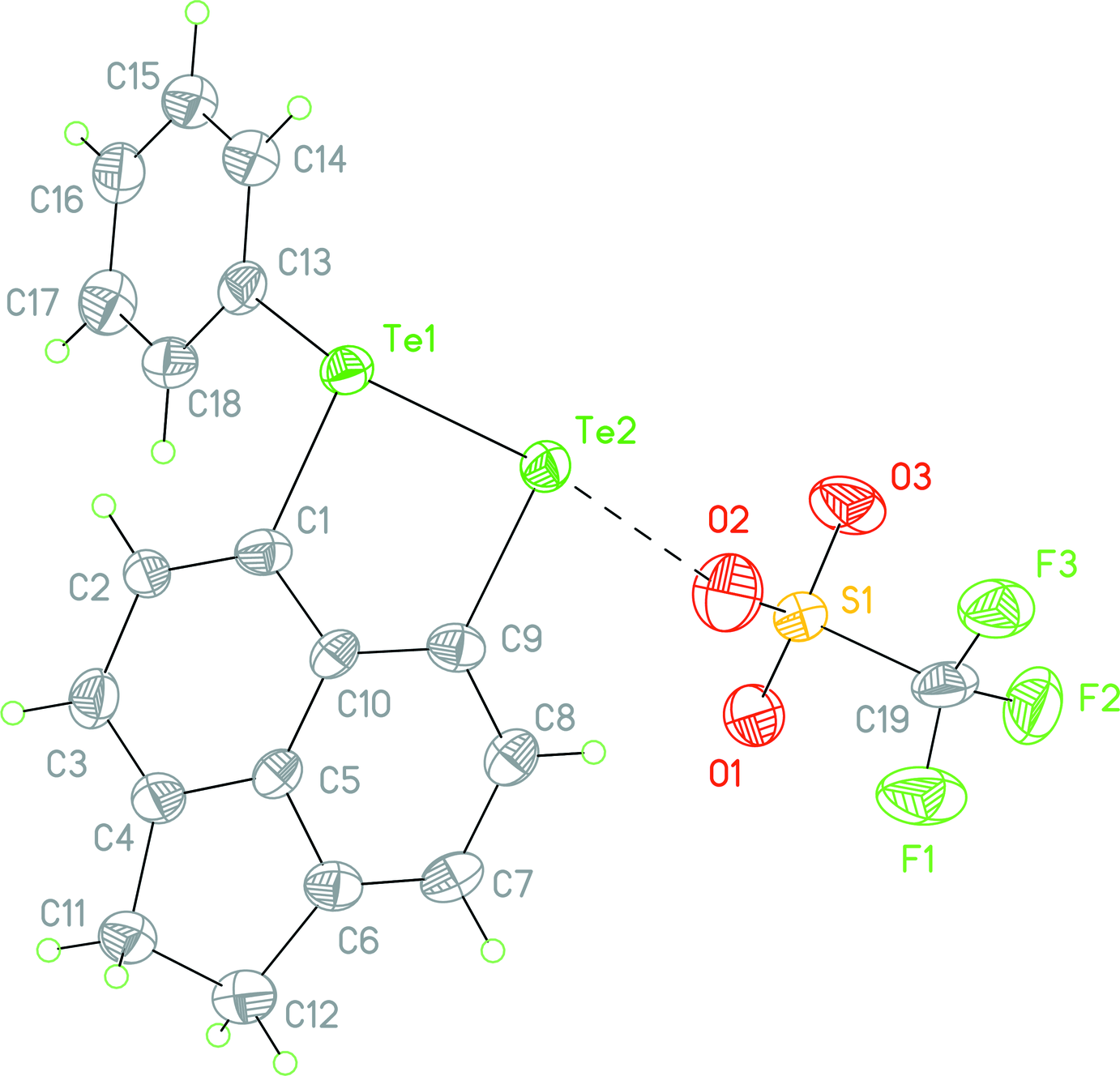

Supplement: Supplementary file 5 [file e-70-o1003-fig1.tif]
